# *RBP3*-Retinopathy—Inherited High Myopia and Retinal Dystrophy: Genetic Characterization, Natural History, and Deep Phenotyping

**DOI:** 10.1016/j.ajo.2023.09.025

**Published:** 2024-02

**Authors:** Michalis Georgiou, Kaoru Fujinami, Anthony G. Robson, Yu Fujinami-Yokokawa, Ahmed F. Shakarchi, Marco H. Ji, Sami H. Uwaydat, Angela Kim, Masha Kolesnikova, Gavin Arno, Nikolas Pontikos, Omar A. Mahroo, Stephen H. Tsang, Andrew R. Webster, Michel Michaelides

**Affiliations:** a From Moorfields Eye Hospital (M.G., K.F., A.G.R., G.A., N.P., O.A.M., A.R.W., M.M.), London, UK; b UCL Institute of Ophthalmology (M.G., K.F., A.G.R.m G.A., N.P., O.A.M., A.R.W., M.M.), University College London, London, UK; c Jones Eye Institute (M.G., A.F.S., M.H.J., S.H.U.), University of Arkansas for Medical Sciences, Little Rock, Arkansas, USA; d Laboratory of Visual Physiology (K.F., Y.F.-Y.), Division of Vision Research, National Institute of Sensory Organs, National Hospital Organization Tokyo Medical Center, Tokyo, Japan; e Department of Health Policy and Management (Y.F.-Y.), Keio University School of Medicine, Tokyo, Japan; f Jonas Children's Vision Care (A.K., M.K., S.H.T.), Departments of Ophthalmology, Pathology & Cell Biology, Columbia Stem Cell Initiative, Columbia University, and Edward S. Harkness Eye Institute, New York–Presbyterian Hospital, New York, New York, USA

## Abstract

**Purpose:**

To examine the genetic and clinical features and the natural history of *RBP3*-associated retinopathy.

**Design:**

Multi-center international, retrospective, case series of adults and children, with moleculraly confirmed *RBP3*-asociated retinopathy.

**Methods:**

The genetic, clinical, and retinal imaging findings, including optical coherence tomography (OCT) and fundus autofluorescence (FAF), were investigated both cross-sectionally and longitudinally. The results of international standard full-field electroretinography (ERG) and pattern electroretinography (PERG) were reviewed.

**Results:**

We ascertained 12 patients (5 female and 7 male) from 10 families (4 patients previously reported). Ten novel disease-causing *RBP3* variants were identified. Ten patients were homozygous. The mean age (±SD, range) of the group was 21.4 years (±19.1, 2.9-60.5 years) at baseline evaluation. All 12 patients were highly myopic, with a mean spherical equivalent of –16.0D (range, –7.0D to –33.0D). Visual acuity was not significantly different between eyes, and no significant anisometropia was observed. Mean best-corrected visual acuity (BCVA) was 0.48 logMAR (SD, ±0.29; range, 0.2-1.35 logMAR); at baseline. Eleven patients had longitudinal BCVA assessment, with a mean BCVA of 0.46 logMAR after a mean follow-up of 12.6 years. All patients were symptomatic with reduced VA and myopia by the age of 7 years old. All patients had myopic fundi and features in keeping with high myopia on OCT, including choroidal thinning. The 4 youngest patients had no fundus pigmentary changes, with the rest of the patients presenting with a variable degree of mid-peripheral pigmentation and macular changes. FAF showed variable phenotypes, ranging from areas of increased signal to advanced atrophy in older patients. OCT showed cystoid macular edema at presentation in 3 patients, which persisted during follow-up in 2 patients and resolved to atrophy in the third patient. The ERGs were abnormal in 9 of 9 cases, revealing variable relative involvement of rod and cone photoreceptors with additional milder dysfunction post-phototransduction in some. All but 1 patient had PERG evidence of macular dysfunction, which was severe in most cases.

**Conclusions:**

This study details the clinical and functional phenotype of *RBP3*-retinopathy in the largest cohort reported to date. *RBP3*-retinopathy is a disease characterized by early onset, slow progression over decades, and high myopia. The phenotypic spectrum and natural history as described herein has prognostic and counseling implications. *RBP3*-related disease should be considered in children with high myopia and retinal dystrophy.

The *RBP3* gene encodes for the interstitial retinoid binding protein (IRBP; Online Mendelian Inheritence in Man (OMIM): 180290), a glycoprotein expressed by photoreceptors and the pineal gland.^1^ Expression of *RBP3* by rod and cone photoreceptors is transactivated by the transcription factor cone–rod homeobox (CRX).^2^ *CRX* is a well-established gene associated with retinal dystrophy.^3^ IRBP is the most abundant protein found in the interphotoreceptor space, the extracellular space between the photoreceptor outer segments and the retinal pigment epithelium (RPE).^4^ RBP3 is a large, 135-kDa secreted protein that binds and transports *cis*/*trans* retinols between photoreceptors and RPE.^4^ The irbp^–^/^–^ knock-out (KO) mouse shows abnormalities of photoreceptor morphology and significantly reduced photoreceptor survival and electroretinogram (ERG) responses, with slow progression over time.^5^ Exaggerated eye growth has been reported in IRBP-deficient mice in early development.^6^

*RBP3*-retinopathy has been reported in only 3 families, with a total of 8 affected members. den Hollander *et al* reported 4 adult siblings from a consanguineous Italian family with autosomal recessive retinitis pigmentosa (arRP) and *RBP3*-variants.^7^ In the second report of *RBP3* variants causing human disease, Arno *et al* reported the clinical phenotypes in 4 children from 2 families, with homozygous nonsense variants, with generalized rod and cone dysfunction and unremarkable fundus appearance.^8^ All 8 children had high myopia, highlighting *RBP3*-variants as a rare cause of retinal dystrophy and high myopia.^7^^,^^8^

The natural history and visual prognosis of *RBP3*-retinopathy is poorly understood, and informed patient management is limited by the rarity of the disease and related literature. Herein we examine the clinical characteristics along with the structural and functional outcomes in the largest *RBP3* cohort reported to date, consisting of 12 molecularly confirmed children and adults. We describe their genetic, clinical, and electrophysiologic features; investigate genotype–phenotype correlations; and establish longitudinal clinical correlations among best-corrected visual acuity (BCVA) and age, optical coherence tomography (OCT) characteristics, and fundus autofluorescence (FAF) features.

## METHODS

This retrospective case series study adhered to the tenets of the Declaration of Helsinki. Each subject (and a parent for subjects <18 years of age) gave written informed consent before genetic testing. The study is in agreement with local institutional ethics committees.

### SUBJECTS

Adults and children with *RBP3*-retinopathy who were examined in the retinal genetics service in 3 tertiary centers (Moorfields Eye Hospital, London, UK; Edward S. Harkness Eye Institute, New York–Presbyterian Hospital, New York, NY, USA; and National Hospital Organization Tokyo Medical Center, Tokyo, Japan), were recruited. *RBP3*-retinopathy diagnosis was based on clinical findings and family history, and was confirmed by genetic testing in all patients.

### *RBP3* GENETIC ANALYSIS

A combination of direct Sanger sequencing and next generation sequencing, including panels of retinal dystrophy genes, whole exome sequencing (WES), and whole genome sequencing (WGS), was used to identify variants in *RBP3*. All recruited patients were reassessed for their detected *RBP3* variants, as described in the Supplemental Material (Methods).

### OCULAR EXAMINATION AND BCVA

Review of clinical records, including medical and ocular histories, slitlamp biomicroscopy, and dilated funduscopic examination was performed. Age of onset was defined as the age at the first reported symptoms. BCVA was measured using Snellen charts and converted into logarithm of the minimum angle of resolution (logMAR) units for statistical analysis. BCVA of the best-seeing eye was used to categorize patients into 1 of 4 groups based on the World Health Organization (WHO) visual impairment criteria, which defines a person with no or mild visual impairment when presenting VA is ≤0.48 logMAR, moderate impairment when VA is 0.48 to 1 logMAR, severe if it is 1 to 1.3 logMAR, and blindness if it is >1.3 logMAR. Low vision corresponds to patients with moderate and severe impairment.

### RETINAL IMAGING

Fundus photography (Optos ultra widefield camera; Optos or Topcon), infrared reflectance (IR), spectral domain (SD)–OCT and short-wavelength (488-nm) FAF were performed longitudinally for most of the patients. Analysis was performed using all available data. Not all modalities or tests were available at all visits, and different baseline and last follow-up were used to maximize follow-up time for all the studied parameters. The mean age and follow-up time are reported individually for each parameter. The presence of complications due to high myopia were also reviewed. Qualitative and quantitative imaging analysis were attempted for FAF and OCT scans.

### ELECTROPHYSIOLOGICAL TESTING

Full-field electroretinograpy (ERG) and pattern electroretinograpy (PERG) testing were performed, incorporating the standards of the International Society for Clinical Electrophysiology of Vision (ISCEV).^9^^,^^10^ PERG P50 was used as an objective measure of macular function and the dark-adapted (DA) and light-adapted (LA) full-field ERGs used to assess generalized rod and cone system function. The ERG data were compared with a reference range from a group of healthy subjects (age range, 10-79 years).^11^^,^^12^ The amplitudes of the main full-field ERG components were plotted as a percentage of the age-matched lower limit of normal (defined as the 5th percentile), whilst peak times were plotted as the difference from the age-matched upper peak time limit (95th percentile), including the DA 10 ERG a-wave, and the LA 3 single flash ERG b-wave and the LA 30-Hz ERG.

### STATISTICAL ANALYSIS

Statistical analysis was carried out using SPSS Statistics for Windows (version 22.0; IBM Corp.). Significance for all statistical tests was set at *P* < .05. The Shapiro–Wilk test was used to test for normality for all variables.

## RESULTS

### DEMOGRAPHIC DATA

We ascertained 12 patients (5 female and 7 male), from 10 families. Four patients (P1-P4) were previously reported.^8^ Clinical data and BCVA were available at 1 or more visits for all patients. The mean age (±SD, range) of the group was 21.4 years (±19.1, 2.9-60.5 years) at first evaluation. The baseline age and the follow-up time is indicated below for each individual assessment. Table 1 summarizes the demographics, age at first examination, follow-up time, and BCVA for each patient.

**TABLE 1:** Demographic and Clinical Characteristics of Study Patients

Patient	Sex	Race	Age (y)	Baseline BCVA	Follow-up Time (y)	Final BCVA	Mean Spherical Equivalent (D)
P01	M	Turkish	6.04	0.45	15.61	0.30	–24.63
P02	F	Turkish	4.88	0.40	15.61	0.30	–14.25
P03	M	Afghan	2.86	0.50	13.75	0.24	–13.13
P04	M	Afghan	5.15	0.30	9.78	0.06	–7.00
P05	M	Kurdish/Iraqi	20.61	0.60	11.09	0.84	PCIOL
P06	F	Pakistani	14.04	0.20	7.94	0.30	–11.75
P07	F	NA	33.66	0.41	9.78	0.74	–13.50
P08	M	Pakistani	16.65	0.44	6.77	0.63	–11.00
P09	F	Bangladesh	30.07	0.39	7.82	0.54	–32.00
P10	F	NA	56.00	0.51	1.00	0.54	PCIOL
P11	M	NA	60.50	1.35	NA	NA	PCIOL
P12	M	Japanese	6.00	0.20	39.00	0.57	–17.00

BCVA = best corrected visual acuity (in logMAR, presented as mean for right and left eyes); D = diopters; NA = not available; PCIOL = posterior chamber intraocular lens.

### GENETIC ANALYSIS

**FIGURE 1:**
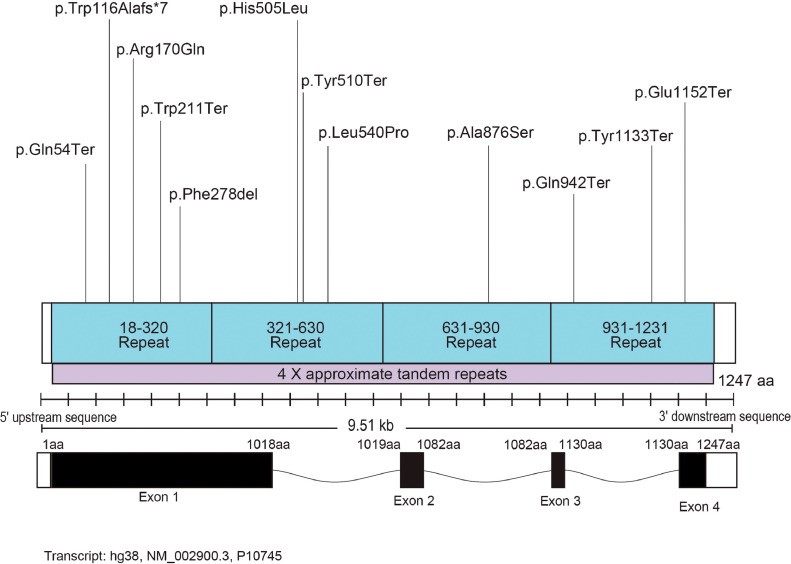
Structure of the *RBP3* genomic locus. The identified variants in the current cohort are shown.

All recruited patients had likely pathogenic variants or variants of uncertain significance in the *RBP3* gene. A total of 12 variants were identified, with only 2 of them previously reported.^8^ Ten patients were homozygous for the identified variants. Table 2 summarizes the genetics of all patients, and Figure 1 presents the localization of the identified variants in the gene domains. Eight patients were not previously reported (Table 2). The Supplemental Table presents all sequence variants, based on their HGVS nomenclature (ID: NM_002900.3) and their predicted effect, pathogenicity assessment based on the American College of Medical Genetics and Genomics (ACMG) guidelines, allele frequency, coverage, general and functional prediction scores, and conservation scores.

**TABLE 2:** Genetics of Study Patients

Patient	Pedigree	Variant 1		Variant 2	
		c.DNA	Protein	c.DNA	Protein
P01^a^	GC17452	c.3454G>T	p.E1152*	Homozygous	
P02^a^	GC17452	c.3454G>T	p.E1152*	Homozygous	
P03^a^	GC19774	c.1530T>A	p.Y510*	Homozygous	
P04^a^	GC19774	c.1530T>A	p.Y510*	Homozygous	
P05	GC19334	c.832_834delTTC	p.Phe278del	Homozygous	
P06	GC19393	c.160C>T	p.Gln54*	Homozygous	
P07	GC20317	c.1619T>C	p.Leu540Pro	Homozygous	
P08	GC21328	c.509G>A	p.Arg170Gln	Homozygous	
P09	GC29680	c.3399T>G	p.Tyr1133*	Homozygous	
P10	NY01	c.2824C>T	p.Gln942*	Homozygous	
P11	NY02	c.1514A>T	p.His505Leu	c.2626G>T	p.Ala876Ser
P12	JP01	c.632G>A	p.Trp211*	c.345_349delCTGGC	p.Trp116AlafsTer7

a Previously reported patient variants by Arno et al.^8^

### DISEASE ONSET, SIGNS, AND SYMPTOMS

All patients were symptomatic with reduced VA and myopia by the age of 7 years. Four patients were symptomatic at birth, with 1 patient having congenital nystagmus (P08), and 4 patients (33%) lifelong nyctalopia (age range, 16-56 years). All 12 patients were highly myopic, with a mean spherical equivalent of right and left eye at last refraction of –16.0D (range, –7.0 diopters [D] to –33.0 D). No significant anisometropia was observed. Strabismus was documented in 6 patients (50%), with 2 patients undergoing surgery. One patient had clear lens exchange in both eyes before the age of 30 years, and 2 patients had early cataract extraction and intraocular lens implants before the age of 60 years. Otherwise, the visual axis was clear in all patients.

**FIGURE 2:**
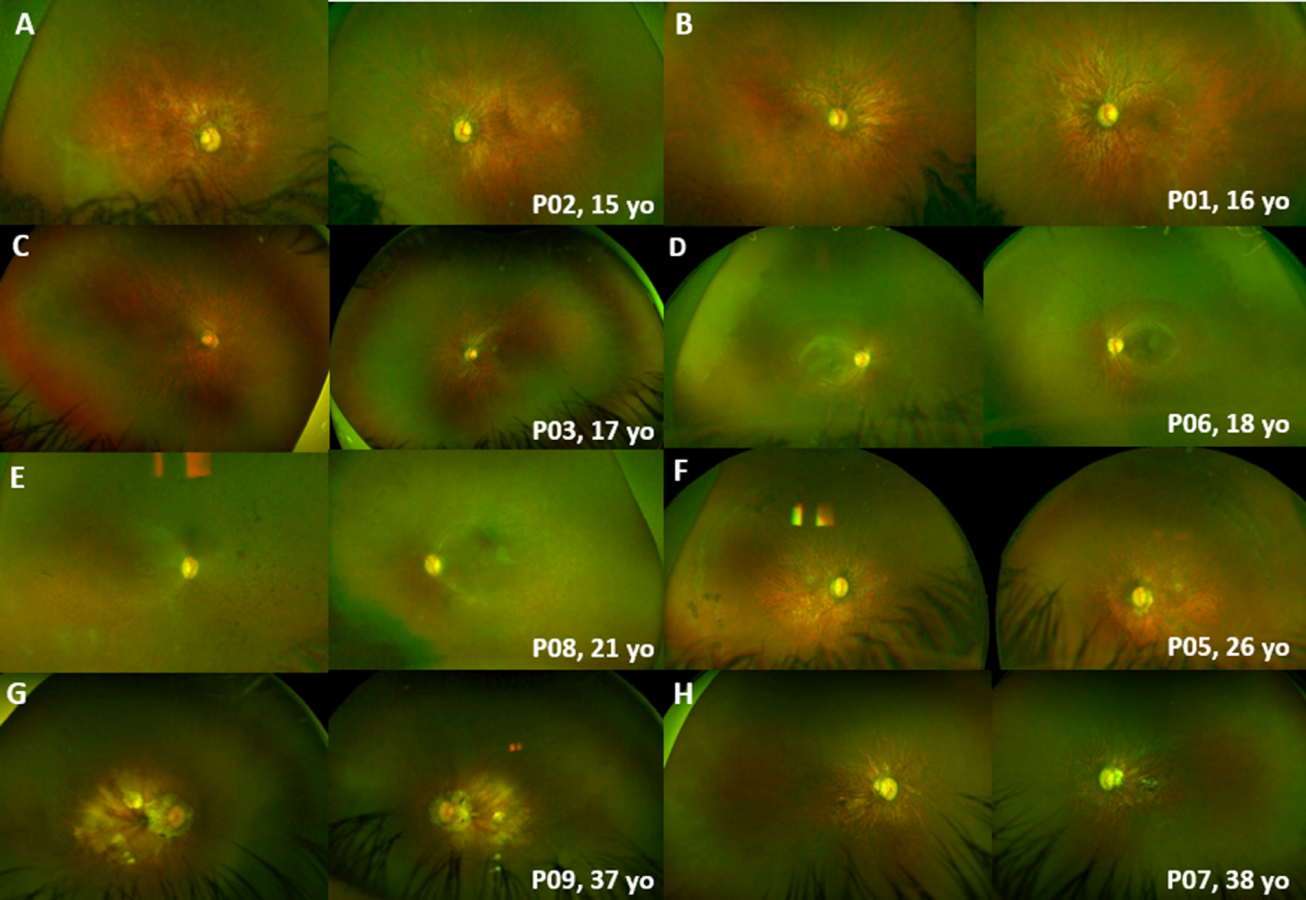
Color fundus photographs of patients with *RBP3*-retinal dystrophy. The fundus had a symmetric appearance in all cases, with myopic changes, including retinal and choroidal thinning, and prominent choroidal vessels. A-C. No fundus pigmentary changes in younger patients. D-F. Variable degree of mid-peripheral pigmentation. F-H. Macular atrophic changes in older individuals. F. Peripheral punched-out chorioretinal atrophy, arranged in multiple curvilinear streaks (Schlaegel-like lines) bilaterally, in P5.

All patients had myopic fundi and features in keeping with high myopia, including retinal and choroidal thinning, and prominent choroidal vessels. The fundus findings were symmetric between the left and right eyes in all cases, and at all visits (Figure 2). The four youngest patients had no fundus pigmentary changes (Figure 2, A-C), with the rest presenting with a variable degree of mid-peripheral intraretinal pigmentation (Figure 2, D-F) and/or macular atrophic changes (Figure 2, F-H). All 4 patients with nyctalopia had pigmentary fundi changes. No patient had documented choroidal neovascularization or retinal detachment due to high myopia. One patient had peripheral punched-out chorioretinal atrophy (P5) arranged in multiple curvilinear streaks (Schlaegel-like lines) bilaterally (Figure 2, F), which was observed over 6 years, with increasing pigmentation over time.

### NON-OCULAR MANIFESTATIONS

No non-ocular manifestations were identified. However, ascertainment bias cannot be excluded, as the vast majority of patients were recruited from a stand-alone eye hospital (Moorfields Eye Hospital).

### VISUAL ACUITY

**FIGURE 3:**
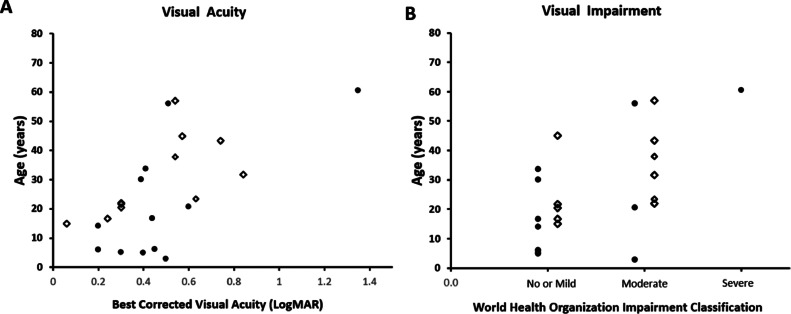
Visual acuity and visual impairment graphs. A. Scatter graph of age and visual acuity for all individuals. B. Stacked scatter plot of visual impairment category per World Health Organization and age. Bullets represent data from baseline evaluation; open diamonds represent data from last follow-up.

BCVA was assessed cross-sectionally and longitudinally. Baseline BCVA was highly variable among patients, but there was no significant interocular difference (W = 10, Wilcoxon signed rank test). Mean BCVA of the right and left eyes was 0.48 logMAR (SD, ± 0.29; range, 0.2-1.35 logMAR) at baseline (mean age, 21.4 ± 19.1 years; range, 2.9-60.5 years). Eleven patients had longitudinal BCVA assessment, with a mean BCVA of 0.46 logMAR (SD, ±0.23 logMAR; range, 0.06-0.84) after a mean follow-up of 12.6 years. No patient had any other vision limiting disease. Figure 3A, shows the distribution of mean BCVA with age, both at baseline and at follow-up. Based on the WHO visual impairment criteria, 8 patients (67%) had no or mild visual impairment, 3 patients (25%) had moderate impairment, 1 (8%) had severe impairment, and no patient was blind. In total, 4 patients (33%) had low vision. Figure 3B, depicts the age distribution for each class of visual impairment at the initial and latest visit.

### FUNDUS AUTOFLUORESCENCE

**FIGURE 4:**
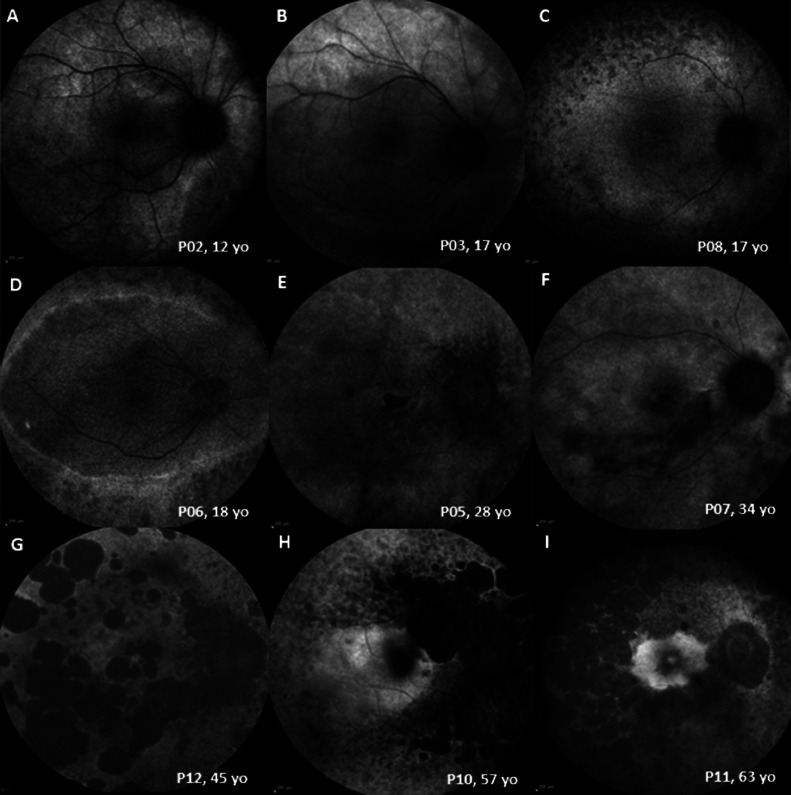
Fundus autofluorescence (FAF) patterns in *RBP3*-retinopathy, with older individuals showing a greater degree and extent of atrophy.

FAF was available for all 12 patients for cross-sectional assessment (mean age ±SD, 27.0 ± 18.6 years; range, 4.7-61.3 years). FAF showed variable phenotypes, ranging from areas of increased signal to advanced atrophy in older patients (Figure 4). The younger patients with relatively normal fundus appearance showed areas of increased signal on FAF (Figure 4, A-B). All patients after the third decade of life showed areas of atrophy within the temporal vascular arcades (Figure 4, E-I). Patients after the fourth decade developed extensive peripheral atrophic changes (Figure 4, G-I). FAF findings were symmetric between eyes. Eleven patients had longitudinal FAF assessment, with a mean follow-up of 6.4 years (range, 0.4-11.9 years). Three patients (age range, 21-38 years) had progressive FAF abnormalities over a mean follow-up of 8.2 years.

### OPTICAL COHERENCE TOMOGRAPHY

**FIGURE 5:**
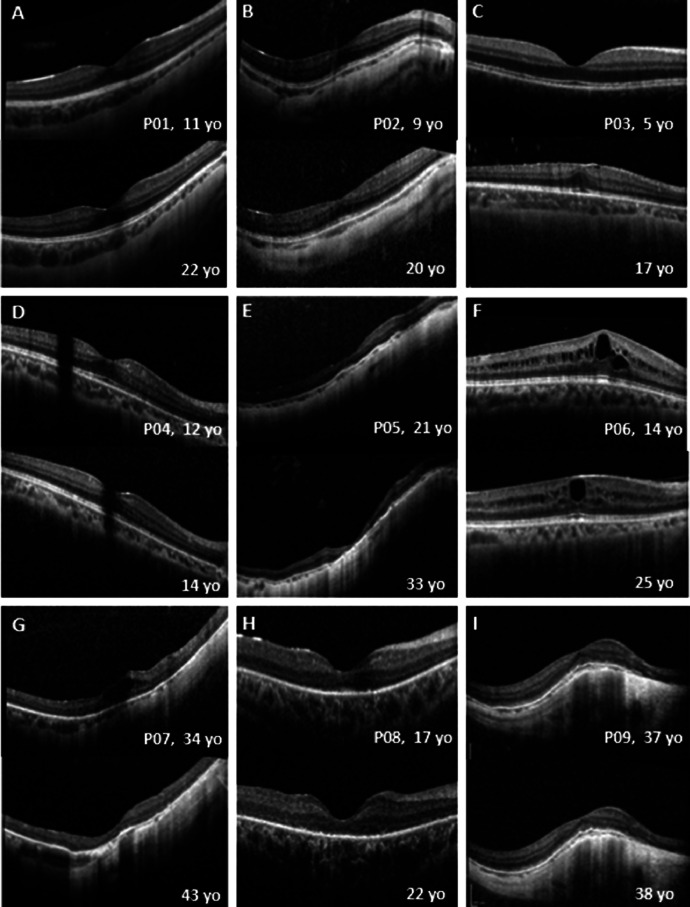
Longitudinal assessment of optical coherence tomography (OCT) in *RBP3*-retinopathy. A-I. Transfoveal horizontal OCT of the right eye at baseline (top) and at follow-up (bottom). Ages at baseline and follow-up are indicated. All patients have increased reflectivity of the retinal pigment epithelium (RPE) and choroidal thinning. Patients 1, 2, 3, 4, and 6 (A, B, C, D, and F) show a continuous ellipsoid zone (EZ) that remains stable over the follow-up period. Patient 5 (E) showed atrophic changes and thinning at baseline, which worsened over follow-up. Patient 6 (F) had cystoid macular edema (CME) at baseline, which persisted over follow-up. Patient 7 (G) had CME at baseline, which resolved during follow-up with worsening atrophic changes and retinal thinning. Patients 8 and 9 (H and I) had EZ disruption, which worsened during follow-up centrally and peripherally, respectively. yo = years old.

OCT was available for all patients at a mean age of 27 years (range, 4.7-60.6 years), and 11 patients had longitudinal data with a mean follow-up of 6.8 years (range, 0.5-11.9 years). All patients had increased reflectivity of the RPE and choroidal thinning. OCT showed cystoid macular edema (CME) at presentation in 5 eyes of 3 patients (Figure 5, F-G), which persisted during follow-up in 3 eyes (Figure 5, F) and resolved in 2 eyes (Figure 5, G). Five patients (mean age, 10.2 years; range, 4.7-14.04 years) showed a continuous ellipsoid zone (EZ) that remained stable over a mean follow-up period of 9.2 years. The length of the preserved EZ was not quantified because of the variable imaging systems and the lack of correction for axial length, given also the high myopia of the cohort. Four patients with a mean age of 40.3 years (range, 21.1-60.6 years) had atrophic changes, with 2 worsening over a follow-up of 10.3 years. Three patients (mean age, 37.9 years; range, 17-57 years) had EZ disruption, either centrally or peripherally, that worsened over time. A variable degree and extent of epiretinal membrane (ERM) was observed in 9 patients (75%). Examples of longitudinal OCT assessment are presented in Figure 5.

### ELECTROPHYSIOLOGY

**FIGURE 6:**
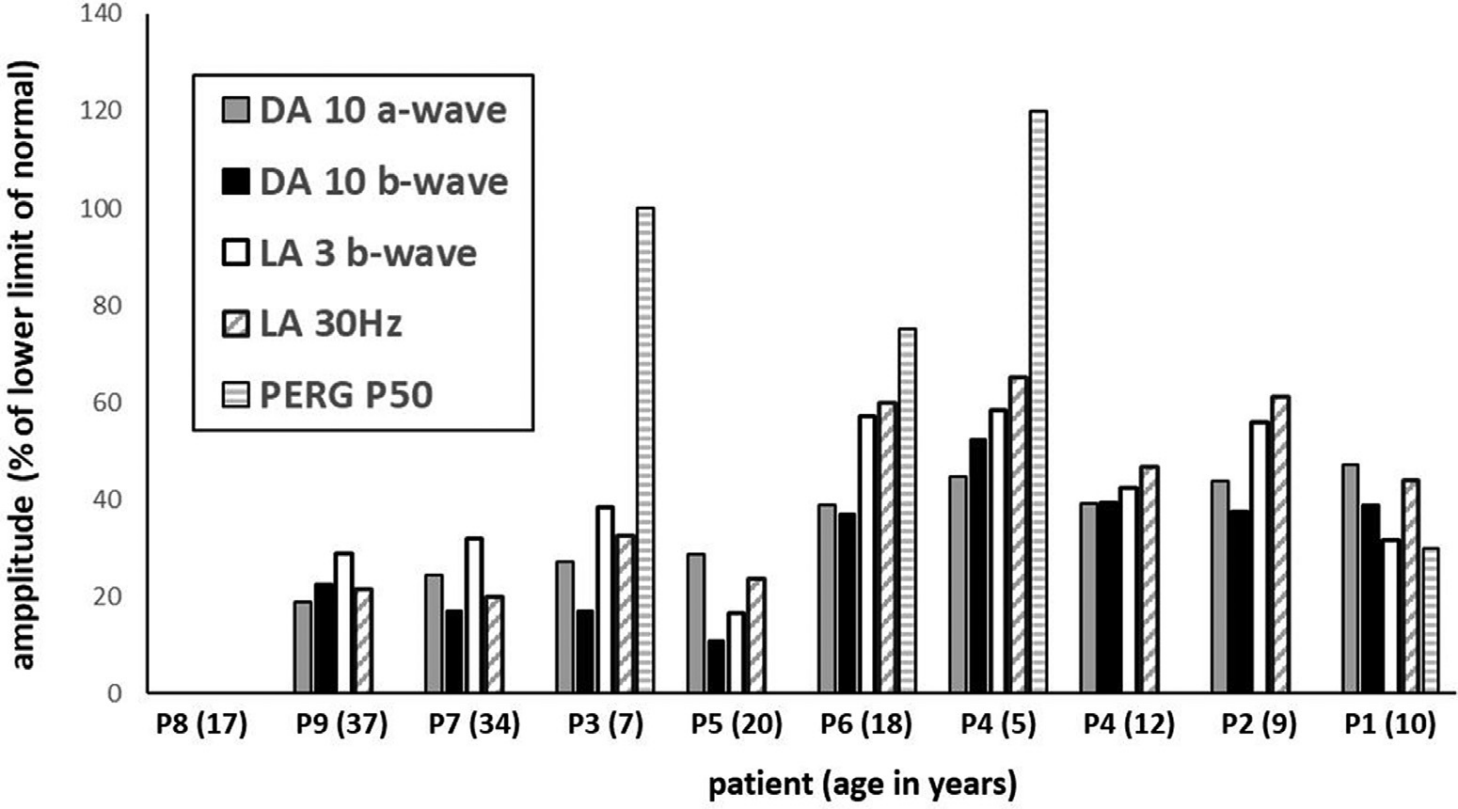
Electrophysiology assessment. The main full-field electroretinography (ERG) findings are summarized in 9 subjects tested according to the International Society for Clinical Electrophysiology of Vision (ISCEV) standard methods. The amplitudes of the dark-adapted (DA) 10 ERG a-waves and b-waves, light-adapted (LA) 30-Hz ERG, and LA 3 ERG b-waves are plotted as a percentage of the age-matched lower limit (5^th^ centile) of the (“normal”) reference range, with values arranged in ascending order of DA10 ERG a-wave amplitude, with exception of baseline data in P4 (included to compare with follow-up data in the same subject). Note that ERGs were undetectable in P8. Patients are identified along the abscissa (age at time of testing in parentheses).

Nine patients (age range 5-37 years; median 17 years) underwent ERG and PERG testing incorporating the ISCEV standard protocols and were recorded with gold foil corneal electrodes. There was a high degree of interocular ERG symmetry based on amplitudes of the DA 0.01, DA 10 a- and b-waves, the LA3 a- and b-waves, and LA 30 Hz ERGs (slope = 0.99, *r^2^* = 0.97). The full-field ERG findings in right eyes are summarized in Figure 6, including baseline and follow up data from P4.

The DA and LA ERGs were undetectable in 1 case (P8; 17 years of age), consistent with a severe photoreceptor dystrophy. In others, baseline recordings revealed either a similar degree of generalized rod and cone system dysfunction (n = 4) or evidence of a rod–cone pattern of dysfunction (n = 3; P2, P4, and P6). In addition to DA10 ERG a-wave reduction in all cases, indicating rod photoreceptor dysfunction, the DA ERG b:a ratio was subnormal in 3 cases (P3, P5, and P7) and in 7 of 8 the LA3 ERG b:a ratio was subnormal (n = 3; P3, P5, and P6) or borderline (n = 4; equal to or minimally lower than the 5^th^ percentile; P1, P2, P4, and P9). In case P5, with markedly subnormal DA10 ERG a-wave and additional b:a ratio reduction, the b-wave was additionally of short peak time, and the waveform was likely mediated by dark adapted cones (see Discussion). There was no significant correlation or consistent trend between decreasing refractive error and DA10 ERG b:a ratios (RE: y = –0.011x, *r^2^* = 0.14; LE y = 0.026, *r^2^* = 0.22) or LA3 ERG b:a ratios (RE; y = –0.0054, *r^2^* = 0.026; LE y = 0.011, *r^2^* = 0.035).

The LA30 Hz ERGs were delayed in 8 of 8 cases (by 3 to 12 milliseconds), with a strong positive association between age and LA 30Hz peak time delay (*r^2^* = 0.85). PERG P50 was undetectable in most (n = 5), showed significant reduction (n = 2; P1 and P6, 10 and 18 years of age, respectively) and was normal in 2 cases (P3 and P4; 7 and 5 years of age, respectively), consistent with sparing of macular function. Subject P4 was initially tested at the age of 5 years and underwent follow-up recordings at the age of 12 years (ERG amplitudes are quantified and compared in Figure 6). There was initial evidence of a rod–cone dystrophy with normal PERG in keeping with preserved macular function, but 7 years later the ERGs showed more marked worsening of LA than DA ERGs (including an increase in LA30Hz peak times from 31 to 35 milliseconds) and a transition from a rod–cone to similar severity of rod and cone dysfunction, with PERG P50 abolition indicating severe worsening of macular function.

### GENOTYPE–PHENOTYPE CORRELATIONS

Genotype–phenotype assessment was limited by the relatively small number of cases. Half of the patients with milder fundus changes, better BCVA, and best preserved rod function (P1-P4; 5-10 years of age at the time of ERG) were homozygous for null variants (P03-P04), and half were homozygous for non-null variants (P01-P02). These included 3 of the 4 patients with a normal (P3, P4) or detectable (P1) PERG consistent with normal or relatively spared macular function (Figure 6). Three patients with 2 non-null variants (P07, P08, and P11) had more severe disease, including the only patient with undetectable ERGs (P8; tested at the age of 17 years) and another case with a severe ERG phenotype (P7; age 34 years). P11 with non-null variants was the oldest in the cohort, with the most advanced atrophic changes and the worst vision (ERG not performed). The data, based on few cases, suggest that there is a lack of genotype–phenotype correlation and that age may be a more important determinant of disease severity.

## DISCUSSION

The current study describes the genetic, clinical, and imaging characteristics of 12 patients with molecularly confirmed *RBP3*-retinopathy, including cross-sectional and longitudinal analysis. It represents the largest cohort to date to undergo detailed investigation, including multimodal imaging, international standard electrophysiology, and genotype–phenotype assessment. Our results provide insights into the retinal phenotype and natural history, over a wide range of ages. Eight novel sequence variants are reported.

The natural history of the disease is characterized by slowly progressive retinal degeneration affecting both the periphery and the fovea in older individuals. The disorder is characterized by variable relative dysfunction of rod and cone photoreceptors, with a high incidence of milder dysfunction that might be post-phototransduction or inner retinal. The 4 young patients previously reported by our group and included in the current study (P1-P4) had relatively mild disease compared with older individuals, suggesting a strong association between age and disease severity. However, despite 1 case (P4) showing worsening cone function over 7 years, these individuals showed no imaging evidence of progressive retinal degeneration , despite nullizygous variants, suggesting wide phenotypic variability and the possibility of less progressive disease. In the current study, 3 patients had progressive FAF changes with macular involvement during their follow-up period. Patients with nyctalopia had mid-peripheral pigmentary changes. Less pigment migration is consistent with RBP3 preferential role in RPE.^13^ Older patients had advanced atrophic changes and vision loss. The lack of genotype–phenotype correlation and disease variability highlights the need for comprehensive phenotyping. Longitudinal studies over longer follow-up will be required to establish the variability in later life.

Full-field ERGs revealed similar severity of rod and cone system dysfunction in most cases, with marked macular dysfunction similar to that seen in forms of cone–rod dystrophy, although there were exceptions including 1 case with a normal PERG. Three patients had greater rod than cone involvement, including 2 patients with relatively preserved macular function, in keeping with common forms of rod–cone dystrophy. It is highlighted, however, that baseline ERGs in one of these cases (P4) at the age of 5 years progressed over 7 years, revealing a transition to more of a cone–rod functional phenotype (Figure 6). Other *RBP3* cases that present with a rod–cone pattern of dysfunction may evolve similarly. One case with a rod–cone dystrophy showed DA10 ERG a-wave reduction and a low b:a ratio but with shortening of b-wave peak time and with the waveform likely representing a dark-adapted cone system contribution exposed in the absence of rod function, as in some other predominant rod photoreceptor disorders.^14^, ^15^, ^16^, ^17^ In addition to primary photoreceptor dysfunction, most cases showed a low or reduced ERG b:a ratio consistent with additional but milder inner retinal dysfunction. The cause is uncertain, but similar findings have been reported in other photoreceptor and cone–rod dystrophies,^18^, ^19^, ^20^, ^21^, ^22^ including those related to *CRX* variants.^23^, ^24^, ^25^ RBP3 is transactivated by CRX; however, unlike *CRX* cases, *RBP3*-related retinal disease is associated with high myopia. Axial lengths were not available for the current *RBP3* cohort, but there was no evidence that refractive index influenced the ERG b:a ratios, broadly in keeping with previous ERG studies showing a preserved b:a ratio across a wide range of myopic patients.^26^, ^27^, ^28^, ^29^, ^30^

Twelve *RBP3* variants were identified in the current study, including 4 missense (non-null), 4 nonsense (null), 2 truncating variants that may escape nonsense-mediated mRNA decay (NMD; non-null), 1 frameshift (null), and 1 in-frame deletion (non-null). Presumably, loss of function is the genetic mechanism of *RBP3*-retinopathy, given the confirmed autosomal recessive inheritance and the phenotypic features of *irbp^−/−^* KO mouse (photoreceptor abnormality) and *irbp^−/+^* heterozygote mouse (no changes).^5^ The presence of non-null variants may suggest hypomorphic or milder clinical effects; however, the genotype–phenotype correlation based on the presence of null variants was not revealed in the current study. In a previous experimental report, D1080N stopped the secretion of IRBP, creating large insoluble complexes through disulfide bonds, then caused stress within the endoplasmic reticulum (ER) and protein misfolding.^31^ These suggest both loss of function and gain of a harmful function, which may occur in *RBP3*-retinopathy. Although this hypothesis cannot fully support the pathogenicity of the detected missense variants in the current study, further knowledge of clinical and experimental studies could clarify the exact clinical effect of each detected variant. Seven of 12 variants were classified into variants of uncertain significance according to the ACMG guideline. The assessment results are inconclusive, as additional factors could be met in future studies or by accumulating knowledge; PS3, PP1, and PP4. Reassessing these variants with further clinical and experimental evidence could enrich the pathogenicity evaluation of the detected variants in such a rare disorder. The multiple roles of RBP3 in the retina are poorly understood. Elevated expression of photoreceptor-secreted RBP3 may have a role in protection against the progression of diabetic retinopathy due to hyperglycemia by inhibiting glucose uptake via GLUT1 and decreasing the expression of inflammatory cytokines and vascular endothelial growth factor (VEGF).^32^ The *irbp^−/−^* KO mouse shows photoreceptor degeneration, and markedly reduced rod and cone responses measured by ERG by 1 month of age. However, there is only slow deterioration in rod function thereafter. Decreased secretion of RBP3, resulting in increased expression of inflammatory cytokines and VEGF, may be related to the persistent CME and the high prevalence of ERM in patients in this series. All reported *RBP3* patients also are highly myopic. In the *irbp^−/−^* KO mouse, axial length is significantly increased with a corresponding marked myopic shift. Collectively, these data support the role of IRBP in normal eye growth and retinal development. High myopia appears to be a hallmark of the *RBP3-*retinal degeneration, and targeted screening of this gene may be considered in patients with high myopia and retinal dystrophy, if not already included in panel screening.

### Future Directions

In total, only 16 patients in 3 studies from 4 centers have been reported in the literature. The disease is likely rare; however, the nonspecific findings in younger patients, such as high myopia and normal fundus, may lead to under-diagnosis of the disease. *RBP3* screening in young patients with pathologic myopia, with or without retinal dystrophy, may further help to estimate the prevalence of the disease, as well as contribute to improved understanding of the disease phenotype and natural history by identifying more affected individuals.

### Limitations

The main limitations of our study are the retrospective design, the lack of a control group. and the variable follow-up duration. Visual field data are lacking. Despite these limitations, this study provides the most comprehensive analysis to date of the genetic, structural, functional, and clinical characteristics *RBP3-*retinopathy, including insight into natural history.

### Conclusions

This study details the clinical and electrophysiological phenotype of *RBP3* retinopathy in the largest cohort reported to date. *RBP3*-retinopathy is a disease characterized by early onset, slow progression over decades, high myopia, and a variable retinal function phenotype. The phenotypic spectrum and natural history as described herein have prognostic and counseling implications. *RBP3*-related disease should be considered in adults and children with high myopia and retinal dystrophy, with or without nyctalopia.

## Appendix Supplementary materials

Image, application 1

Image, application 2
